# The Influence of Subscapularis Muscle Split Location on Subscapularis Function After the Latarjet Procedure

**DOI:** 10.1177/23259671251329516

**Published:** 2025-04-14

**Authors:** Natalia Belotti, Aaron S. Fox, Janina Henze, Richard S. Page, Lukas Ernstbrunner, David C. Ackland

**Affiliations:** * University of Melbourne, Melbourne, Australia; † ETH Zurich, Zurich, Switzerland; ‡ Deakin University, Geelong, Australia; § University Hospital Geelong, Barwon Health, Geelong, Australia; ‖ Department of Orthopaedic Surgery, Box Hill Hospital, Box Hill, Australia; Investigation performed at University of Melbourne, Melbourne, Australia

**Keywords:** upper limb, glenohumeral joint, stability, anterior instability, subluxation

## Abstract

**Background:**

Splitting the subscapularis in the Latarjet procedure is known to influence subscapularis muscle mechanics postoperatively; however, the influence of split level on postoperative muscle and joint function remains poorly understood.

**Purpose:**

To assess the effects of midlevel, lower-third, and upper-third subscapularis split levels in the Latarjet procedure on subscapularis lines of action and moment arms in the shoulder abduction, abduction and external rotation (ABER), and apprehension positions.

**Study Design:**

Controlled laboratory study.

**Methods:**

The Latarjet procedure was performed on 8 fresh-frozen human cadaveric upper extremities with a simulated 20% anteroinferior glenoid bone defect. A midwidth subscapularis muscle belly split was first performed on all specimens in which the conjoint tendon was routed. Lines of action and moment arms of 4 subregions of the subscapularis muscle (superior, mid-superior, mid-inferior, and inferior) were quantified radiographically with the conjoint tendon unloaded and loaded and the glenohumeral joint positioned in (1) 0° of abduction, (2) 90° of abduction, (3) 90° of abduction and full external rotation (ABER), and (4) the apprehension position, defined as ABER with 30° of horizontal extension. Testing was then repeated in random order after rerouting the conjoint tendon through both an upper- and then lower-third subscapularis split. Utmost care was taken to ensure that the subscapularis muscle integrity was not disrupted during the rerouting process.

**Results:**

Subscapularis splitting in Latarjet surgery deformed the muscle fibers below the split level, significantly increasing the inferior inclination of subscapularis muscle lines of action, but only for the midlevel and lower-third subscapularis split levels (*P* < .001). This increased inferior inclination was significantly greater in the ABER and apprehension positions compared with those at 0° and 90° of abduction (*P* < .05). In the ABER and apprehension positions, the adduction moment arms of the mid-superior subscapularis muscle subregion were also significantly larger for the midlevel split compared with the lower-third and upper-third split (*P* < .05), indicating greater depressor capacity.

**Conclusion:**

Latarjet surgery deforms subscapularis muscle fibers below the level of the split, changing subscapularis leverage and line of force. The midlevel subscapularis muscle split in the Latarjet procedure confers greater mechanical advantage in terms of shoulder depressor function and stabilizing potential than that associated with an upper-third or lower-third split, particularly in the ABER and apprehension positions.

**Clinical Relevance:**

Subscapularis muscle leverage and force potential are significantly influenced by split location in Latarjet surgery. A midlevel subscapularis split is likely to provide the greatest mechanical stability, particularly in positions of shoulder instability.

Recurrent anterior shoulder instability is a debilitating condition associated with capsulolabral and osseous damage of both the humerus and glenoid.^9,10,14,26,29^ The Latarjet procedure is an established surgical approach for restoring glenohumeral stability in patients with recurrent anterior shoulder instability and glenoid bone loss. This is primarily achieved by augmentation of the glenoid contact area through the coracoid bone graft and via the conjoint tendon passing through the subscapularis muscle, which creates a sling effect and barrier to anterior subluxation.^22,31^ This sling effect is particularly prominent in the abduction and external rotation (ABER) position, when the anteroinferior joint capsule is tensioned and the risk of anteroinferior translation and ultimately subluxation/dislocation is increased.^16^ Despite its success and long-term recurrence rates between 3% and 8%,^4,14,19-21,25^ the Latarjet procedure remains subject to procedure-specific complications such as graft-related complications,^7,12,17,18^ loss of axial rotation, and subscapularis muscle weakness and fatty infiltration.^13^

During the Latarjet procedure, the coracoid is osteotomized and the bone block with the conjoint tendon attached is transferred through a longitudinal split in the subscapularis muscle belly and secured to the anteroinferior glenoid defect. Screws are then used to secure the graft to the anterior glenoid wall in a traditional flat or congruent arch orientation.^28^ The conjoint tendon contacts and therefore interacts with the subscapularis muscle fibers, because the line of action of the conjoint tendon, which follows the long axis of the humerus, tends to wrap around and deform the subscapularis muscle belly in the vicinity of the split.^5,6,22,27^ Recent biomechanical data suggest that conjoint tendon loading after the Latarjet procedure significantly increases the inferior inclination of the subscapularis and decreases its horizontal flexion leverage.^15^ This suggests that the Latarjet procedure has the potential to alter subscapularis function, which may affect shoulder range of motion^13^ and give rise to changes in joint stability. While the subscapularis split level influences the size of the sling and subscapularis muscle deformation via the conjoint tendon, the effect of different levels of conjoint tendon split on subscapularis muscle function remains poorly understood.

The objective of this study was to evaluate the effect of upper-, mid-, and lower-third subscapularis split levels in the Latarjet procedure on subscapularis lines of action and moment arms of its muscle subregions in the abduction, ABER, and anterior apprehension positions. Because the inferior pull of the conjoint tendon on the subscapularis fibers has been shown to primarily occur at or below the level of the split, we hypothesized that a change in split level would alter the subscapularis lines of action and leverage across its muscle fibers.

## Methods

### Specimen Preparation

Eight fresh-frozen entire upper extremities (4 male, 4 female) were harvested from human cadavers (mean age, 69.3 ± 8.2 years; mean weight, 78.4 ± 4.7 kg) via the university’s Body Donor Program. Specimens were radiographically screened for macroscopic degenerative changes such as glenohumeral joint osteoarthritis, rotator cuff tears, fractures, or previous surgery. This sample size was chosen to achieve a power level of β = .8 (α = .05) for detecting significant differences in moment arm of 5.0 mm and line of action of 10.0° with applied conjoint tendon force.^1-3^ Specimens were thawed at room temperature for 24 hours before testing. The skin and subcutaneous soft tissue proximal to the glenohumeral joint were removed by sharp dissection, and the shoulder musculature was exposed. Three subregions of the deltoid were identified: anterior (clavicular fibers), middle (acromial fibers), and posterior (posterior scapular spine fibers). Additionally, the 4 rotator cuff muscles and the subscapularis muscle were divided into 4 anatomically distinct subregions: superior, mid-superior, mid-inferior, and inferior.^1^ Ethics approval was obtained by the institutional review board.

### Latarjet Surgery

**Figure 1.**
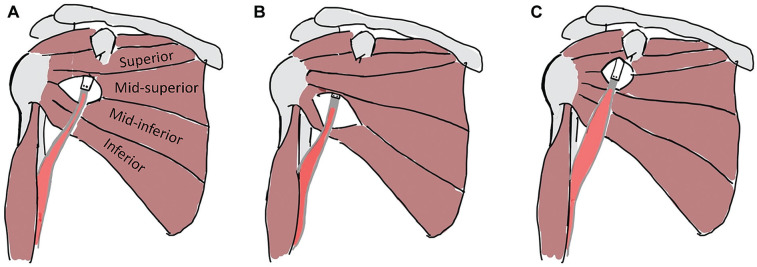
Subscapularis muscle split levels used in the present study, including (A) mid-third, (B) lower-third, and (C) upper-third splits. The conjoint tendon was rerouted through the different subscapularis split levels without altering the bone block position. The subscapularis muscle was divided into 4 equal-sized subregions (fiber bundles), which included the superior, mid-superior, mid-inferior, and inferior subregions.

An anteroinferior glenoid defect was created representing 20% bone loss by surface area of the lower two-thirds of the glenoid surface.^13,16,29^ An oscillating saw was used to create the osteotomy line, which was parallel to the line connecting the supra- and infraglenoidal tubercles. The Latarjet procedure was then performed using the method described previously.^8^ With the aid of bent osteotomes, the coracoid was identified and osteotomized at its base with a mean graft length of 24 mm. A horizontal split of the subscapularis muscle was then performed at the midwidth of the muscle belly, and the glenoid neck was exposed (Figure 1A). The midpoint of the subscapularis muscle-tendon attachment on the scapula was estimated using digital calipers and used as a reference for the horizontal split (Mitutoyo). The coracoid bone block was fixed in a single position at the glenoid plane of the shoulder at 2 to 5 o’clock with 2 cannulated bicortical screws with intraosseous washers and a drilling guide from the Bristow-Latarjet Instability Shoulder System (DePuy Synthes Mitek, Johnson & Johnson). Subsequently, the conjoint tendon was rerouted through different split levels of the subscapularis muscle without changing the bone block position. Finally, the coracoacromial ligament was sutured to the most medial part of the capsule and the capsule was closed.

### Experimental Protocol

**Figure 2.**
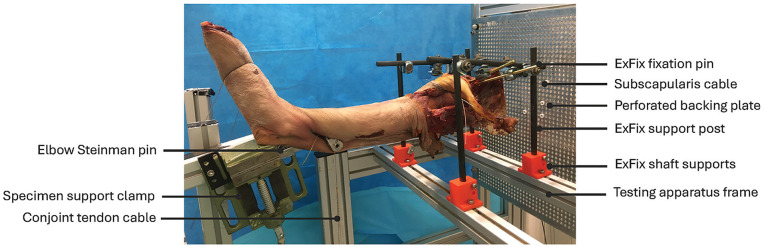
Experimental testing apparatus used for shoulder muscle line of action and moment arm measurements. Specimens were mounted to the testing rig support frame using an external fixator. Four pins passing through the scapula spine were supported by 4 carbon fiber posts mounted to the rig frame using 3-dimensional printed shaft supports. Tensile loads were applied to the shoulder muscle-tendon units using a cable-pulley weight system that applied force along each muscle-tendon unit’s line of action. An additional pulley was secured to the distal humerus and used to apply conjoint tendon loading using a cable and free weight. To reproduce the line of action of each muscle-tendon unit, nylon lines and cables sutured to each distal tendon ran along the muscle belly to an eyelet positioned at the centroid of the origin of each muscle, and then through a perforated plate to a free weight. The upper limb was supported by using a clamp to grasp a Steinman pin inserted into the humerus. Scapular and transverse plane radiographs were taken with a C-arm x-ray fluoroscopy unit. Reproduced with permission from Elsevier.^15^

The moment arms and lines of action of the 4 subscapularis muscle subregions were measured using a custom-designed testing apparatus, and an experimental testing protocol that has been previously validated and assessed for repeatabiliy.^15^ Each scapula was rigidly secured to the apparatus using an external fixation device (DePuy Synthes, Johnson & Johnson) with the glenoid plane aligned with the vertical and the glenohumeral joint initially in the neutral position (Figure 2). Radio-opaque cables were fastened to each subscapularis muscle-tendon unit via its suture while radiolucent nylon lines were then tied to the sutures of the deltoid, supraspinatus, infraspinatus, and teres minor muscle-tendon units. The lines and cables were routed through eyelets at the centroid of each tendon origin on the scapula to a perforated backing plate. A free weight was then attached to each line and cable end to apply tension and simulate physiological loading along each muscle-tendon unit’s line of action. The magnitude of tensile force applied to each muscle-tendon unit and for each task was estimated using a electromyography-driven model as described previously.^23^ The conjoint muscle bellies were released from the humerus and radius, and a nylon line was sutured to its distal tendons with a free weight attached, which applied loading directly to the conjoint tendon.

Each upper limb was positioned passively in joint configurations that represented activities of daily living and positions of glenohumeral joint instability. Placed in random order, the joint positions included (1) 0° of abduction and neutral rotation, (2) 90° of abduction and neutral rotation, (3) 90° of abduction and 90° of external rotation (ABER), and (4) the apprehension position, which was defined as ABER with 30° of horizontal extension. The humerus was immobilized using an external testing frame, and the final glenohumeral joint position was verified radiographically (Fluoroscan InSight 2; Hologic Inc). At each joint position, 0-N, 20-N, and 40-N loads were applied directly to the conjoint tendon,^22,30^ and the glenohumeral joint was radiographed in both the scapular and transverse planes to confirm a congruent glenohumeral joint and to account for radiographic visualization of subscapularis muscle lines of action.

**Figure 3.**
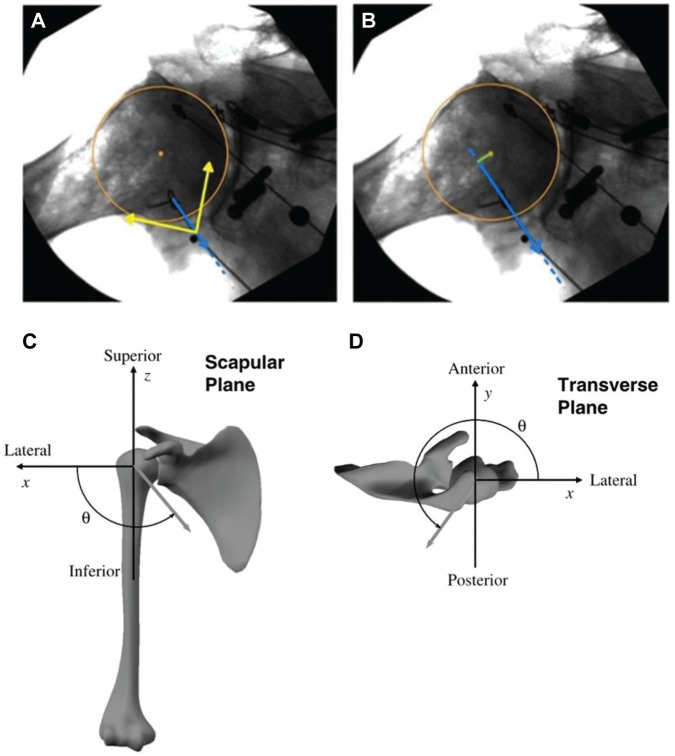
Illustration of subscapularis muscle (A) line of action and (B) moment arm defined using a radiograph taken in the scapular plane, and line-of-action angle definition in the (C) scapular plane and (D) transverse plane. A circle was fit to the humeral head (brown) to define the approximate center of rotation of the glenohumeral joint, while the line of action was digitized using a line segment (blue arrow). A glenoid-fixed coordinate system was defined (yellow arrows) and used to express the orientation of the line of action. The lower subscapularis moment arm was calculated as the perpendicular distance between the line of action and joint center of rotation and indicates adduction capacity. Contact between the conjoint tendon and subscapularis is indicated at the blue arrowhead where the subscapularis line of action deforms. Images C and D reproduced with permission from Wiley^1^

The lines of action of each subscapularis subregion were calculated radiographically from the path of each cable and expressed relative to the glenoid in both the scapular and transverse planes. Each line of action was defined as the unit vector between the point at which the distal muscle-tendon unit loses contact with the humerus and the proximal centroid of origin of that muscle-tendon unit. When a muscle path was deformed or distorted due to conjoint tendon contact, the approximate point at which the muscle path contacted the tendon was taken as the effective proximal attachment point. Lines of action were projected onto the scapular and transverse planes and expressed relative to a glenoid-fixed coordinate system (Figure 3). The moment arm of each muscle-tendon unit with respect to the glenohumeral joint center was measured radiographically using the geometric method.^15^ To achieve this, a geometric circle was fitted to the humeral head in both the scapular and transverse plane images, with this position assumed to approximate the glenohumeral joint center of rotation of the joint based on the anatomic and kinematic landmarks of the glenoid and humerus geometry (Figure 3).

The scapular and transverse plane moment arm of each muscle-tendon unit was defined as the perpendicular distance between its line of action and the joint center of rotation. In the scapular plane, a positive moment arm represented abduction capacity, while a negative moment arm represented adduction capacity. In the transverse plane at 0° of abduction, a positive moment arm represented external rotation capacity while a negative moment arm represented internal rotation capacity; in the 90° of abduction, ABER, and apprehension positions, a positive moment arm represented horizontal extension capacity, while a negative moment arm represented horizontal flexion capacity.

The previous subscapularis split was sutured closed and a lower-third split was then created by dividing the subscapularis muscle fibers, in line with their orientation, between the mid-inferior and inferior subscapularis subregions (Figure 1B). The distal end of the conjoint tendon was then routed through this opening without unscrewing the coracoid, thus maintaining primary stability of the graft. The entire testing protocol was again repeated for an upper-third split, that is, by dividing the subscapularis muscle fibers between the mid-upper and mid-inferior subregions. After testing in the mid-third split configuration, the upper- and lower-third subscapularis split configurations were tested in random order (Figure 1C).

### Statistical Analysis

A 3-way repeated-measures analysis of variance was used to evaluate the influence of the subscapularis split level (upper, mid, and lower), conjoint tendon load (0 N, 20 N, and 40 N), and joint position (0° of abduction, 90° of abduction, ABER, and apprehension) on subscapularis muscle lines of action and moment arms in the scapular and transverse planes after the Latarjet procedure. Standard deviation was used as a measure of data variance, and 95% confidence intervals were calculated with the level of significance set at a *P* value <.05. The Shapiro-Wilk test was used to verify the normality of the sample distribution, and Tukey post hoc tests were used to assess between-group differences.

## Results

### Effect of Joint Position

**Figure 4.**
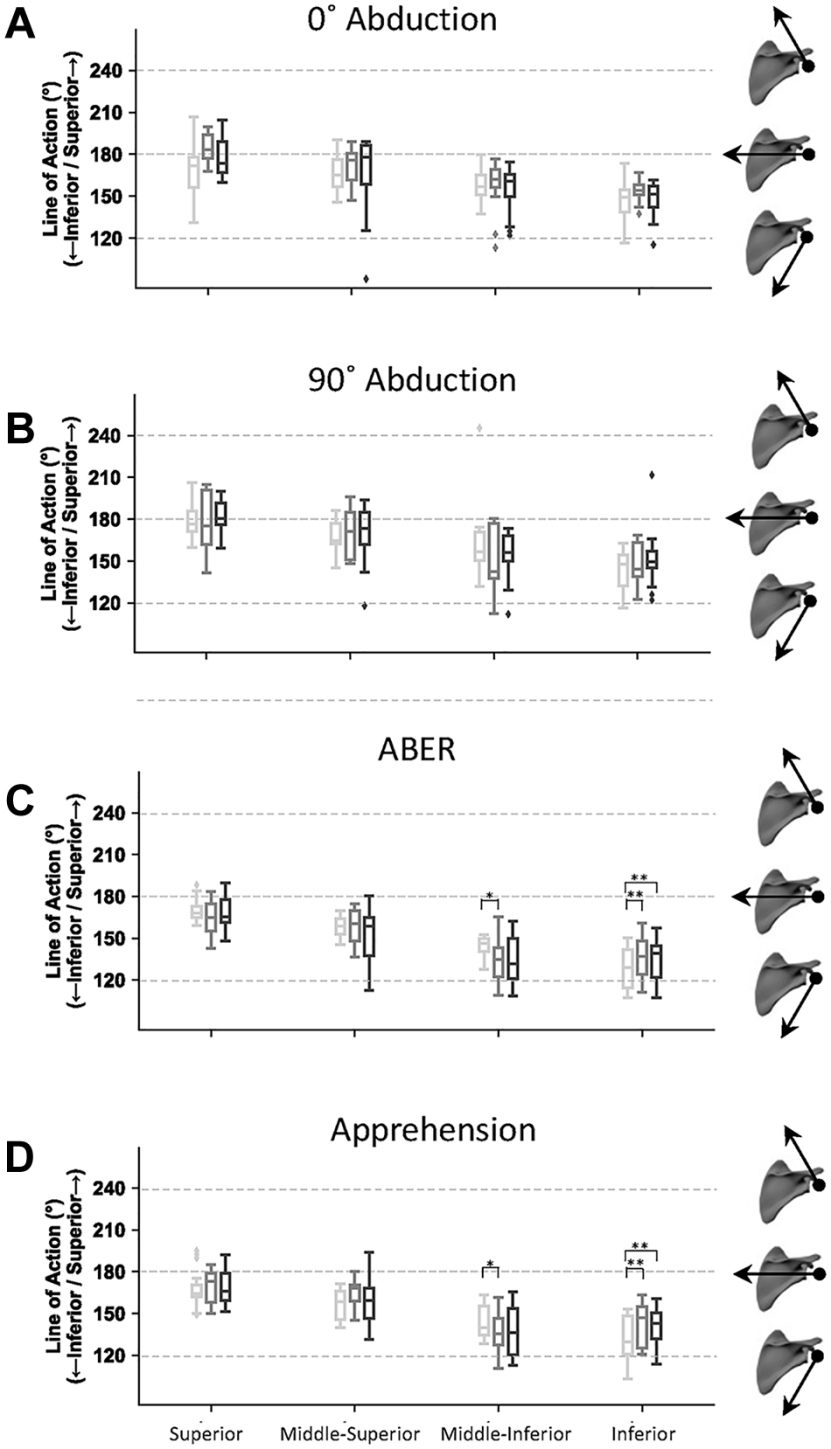
Mean scapular plane lines of action for the superior, mid-superior, mid-inferior, and inferior subscapularis subregions. Given are the lines of action at (A) 0° of abduction, (B) 90° of abduction, (C) abduction and external rotation (ABER), and (D) apprehension in the case of an average conjoint tendon load. Black, dark gray, and light gray bars represent upper-third, midlevel, and lower-third split levels, respectively. Insert scapulae with arrows illustrate line-of-action directions on the vertical axis. *P < .05; **P < .01.

**Figure 5.**
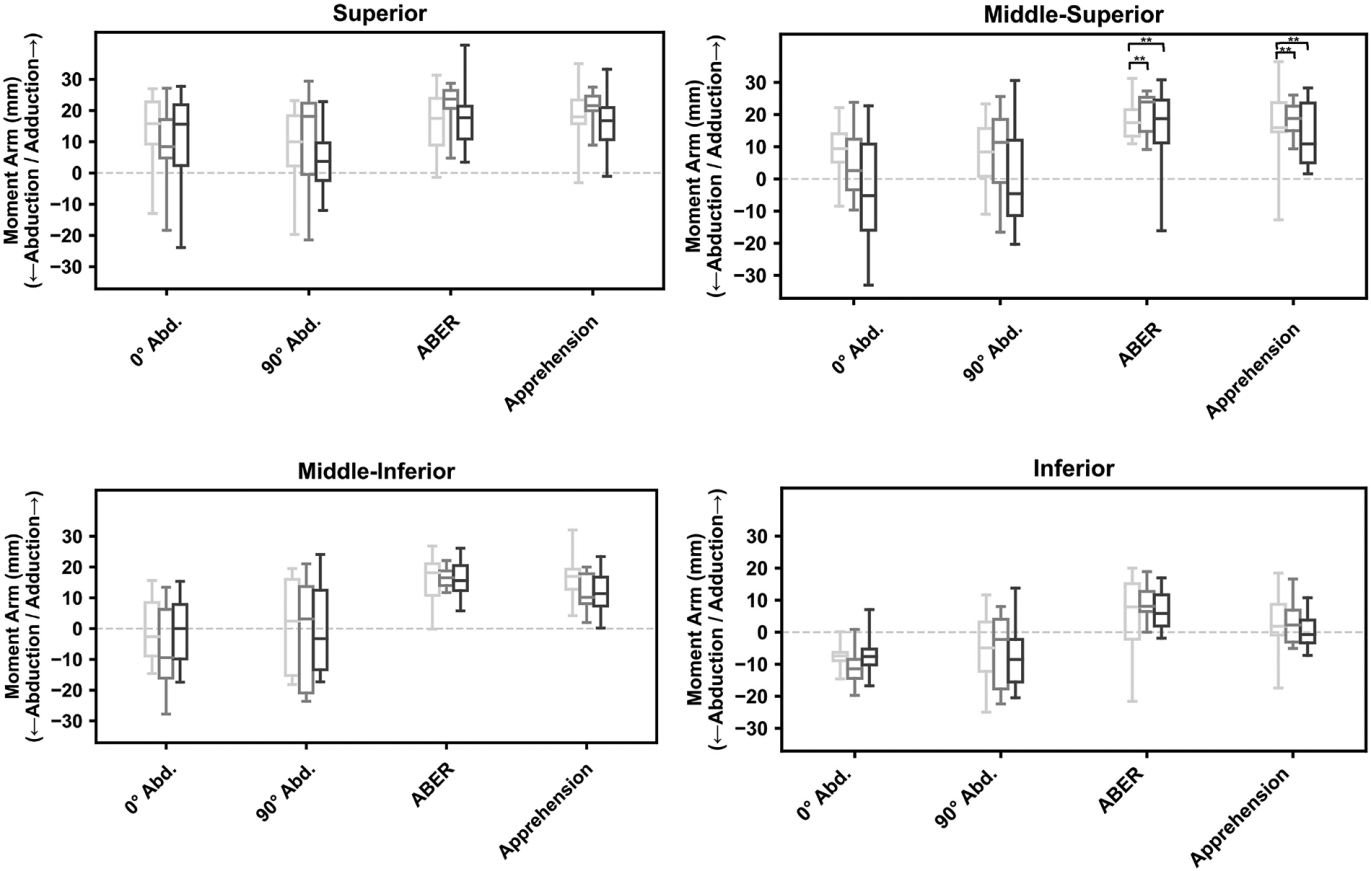
Box plots of mean adduction moment arms for the superior, mid-superior, mid-inferior, and inferior subregions of subscapularis muscle. Given are the moment arms at 0° of abduction, 90° of abduction, abduction and external rotation (ABER), and apprehension in the case of an average conjoint tendon load. Light gray, dark gray, and black boxes represent lower-third, midlevel, and upper-third split levels, respectively. **P < .01. Abd., abduction.

Change in joint position significantly affected the orientation of the lines of action in all subscapularis muscle regions after the Latarjet procedure, that is, the superior, mid-superior, mid-inferior, and inferior subregions (*P* < .001) (Figure 4). In the ABER and apprehension positions, the subregions of subscapularis muscle were more aligned for depressor function and exhibited significantly more inferior inclination relative to those in the 0° of abduction position (Appendix Table A1). In the ABER position, this included the mid-superior (mean difference, 13.8°; 95% CI, 6.5°-20.8°; *P* < .001), mid-inferior (mean difference, 19.9°; 95% CI, 12.9°-26.9°; *P* < .001), and inferior subscapularis (mean difference, 16.2°; 95% CI, 9.9°-22.5°; *P* < .001) subregions. In the apprehension position, significantly more inferior inclination was observed for the mid-inferior (mean difference, 18.5°; 95% CI, 11.4°-25.5°; *P* < .001) and inferior (mean difference, 11.3°; 95% CI, 5.0°-17.6°; *P* < .001) subregions of subscapularis muscle compared with those in the 0° of abduction position. Across all subregions of the subscapularis muscle, there were significantly larger adduction moment arms in ABER and apprehension compared with those at 0° and 90° of abduction (*P* < .001) (Figure 5) (Appendix Table A2).

### Effect of Subscapularis Split Level

Subscapularis split level had a significant effect on the inclination of the mid-inferior and inferior subregions of the subscapularis muscle after the Latarjet procedure for both the ABER and apprehension positions (*P* < .05) (Figure 4). In each case, the split deformed and changed the fiber orientations immediately below the split level, resulting in a more inferior inclination of these muscle fibers compared with those elsewhere in the muscle, with this effect most prominent in the ABER position. For example, the inferior line of action inclination of the mid-inferior subscapularis was significantly greater (more inferiorly inclined) for the midlevel split compared with the lower-third split in the ABER position (mean difference, 10.6°; 95% CI, 5.0°-16.2°; *P* = .024). Similarly, for the apprehension position, the mid-inferior subscapularis subregion was significantly more inferiorly inclined for the midlevel split compared with the lower-third split (mean difference, 8.7°; 95% CI, 3.7°-13.7°; *P* = .002). This effect of significant change in subscapularis line of action immediately below the split level was also observed in the ABER and apprehension positions for the inferior subscapularis subregion. Specifically, the line of action of the inferior subscapularis was significantly more inferiorly inclined for the lower-third split compared with the midlevel or upper-third split (*P* < .05).

In the ABER position, the adduction moment arm and leverage of the mid-superior subscapularis muscle subregion were significantly smaller for the upper-third split compared with the midlevel split (mean difference, 3.7 mm; 95% CI, 1.9-5.5 mm; *P* = .044), with the adduction moment arm for the midlevel split significantly larger than that for the lower-third split (mean difference, 2.6; 95% CI, 0.8-4.4; *P* = .012) (Figure 5). For the apprehension position, the adduction moment arm and leverage of the mid-superior subscapularis muscle subregion were also significantly smaller for the upper-third split compared with the midlevel split (mean difference, 5.6 mm; 95% CI, 3.7-7.5 mm; *P* = .001) and lower-third split (mean difference, 5.1 mm; 95% CI, 1.0-4.2 mm; *P* = .005).

### Effect of Conjoint Tendon Force

An increase in conjoint tendon force was associated with a more inferiorly inclined line of action of the subscapularis parts immediately below the split level, with significant changes observed for the lower-third split across the ABER and apprehension positions (*P* < .05).

## Discussion

This study showed that mid- and lower-third subscapularis splits increase depressor function in the fibers below the split level. This may ultimately increase joint stability by reducing superior subluxation and increasing joint compression via antagonistic co-contraction in concert with the deltoid. Furthermore, there was a greater adduction moment arm for the superior fibers of the subscapularis in the ABER and apprehension positions when a mid-third split was used compared with an upper- or lower-third split. This increased mechanical advantage may be favorable biomechanically and ultimately should be considered intraoperatively while deciding at which level the subscapularis split will be conducted. These findings support our hypothesis of the influence of conjoint tendon force and split level on the inclination of subscapularis lines of action.

We showed that the change in inclination of the subscapularis muscle fibers with the split level was greatest in the fibers below the level of the split after Latarjet surgery. For example, a midlevel subscapularis split resulted in a more inferiorly directed line of action for the mid-inferior subscapularis, while the lower-third split resulted in a more inferiorly directed line of action for the inferior subscapularis. In contrast, an upper-third split altered the inclination of the mid-superior subscapularis, albeit to a lesser degree than the mid- or lower-third splits. The increase in the inferior inclination of the muscle fibers below the level of the subscapularis split is a consequence of the deformation of muscle fibers due to the inferior directed pull of the conjoint tendon. The finding of the midlevel and lower-third split affecting subscapularis muscle lines of action to a greater extent than the upper-third split may be a consequence of the relatively lower number of muscle fibers below the level of the split that are available to resist muscle deformation. That is, a more superiorly positioned split may result in less change in line of action of any given muscle subregion, because more of the subscapularis by muscle volume resides below the split level and resists muscle deformation under the action of the conjoint tendon. While the increased sling size with a more superior positioned subscapularis split may present a greater barrier effect advantage in mitigating joint subluxation in the unstable shoulder, increased contact deformation via the conjoint tendon across more muscle fibers may ultimately present a risk factor for jeopardizing subscapularis integrity.^6,11,13^

The lower-third and midlevel splits increased the inferior inclination of the inferior and mid-inferior subscapularis muscle’s lines of action, which increases the potential of these muscle subregions to produce inferior glenohumeral joint shear. The subscapularis inferior shear potential and depressor function acts as an antagonist to the superior joint shear produced by the deltoid during upper limb function and in resisting subluxation of the humeral head.^2,24^ Therefore, increased subscapularis inferior shear potential may result in increased glenohumeral joint compression forces and ultimately confer greater glenohumeral joint stability. Considering a larger sling with a midlevel split compared with a lower-third split, and potentially greater tension across the anteroinferior joint capsule to prevent anteroinferior subluxation, the findings of the present study lend further support to a midlevel split as providing optimal biomechanical function in the Latarjet procedure, which appears to be most significant in the ABER and apprehension positions where the shoulder is most unstable.

We observed a larger adduction moment arm in the mid-superior subscapularis for the case of a midlevel split relative to an upper-third or lower-third split, with the results significant in the ABER and apprehension positions. This larger subscapularis leverage is due to the muscle path deformation under the action of the conjoint tendon, which increased the distance of the subscapularis muscle fibers to the joint center of rotation, ultimately increasing the torque potential of these muscle fibers. This may increase the capacity of the subscapularis to stabilize the head of the humerus against the glenoid via antagonistic co-contraction against the deltoid’s abductor function.^23^ The finding of greater subscapularis adduction leverage and stabilizing potential for a midlevel split was observed in the ABER and apprehension positions, when the joint is in a position of instability. Future studies ought to consider the effect of split location on subscapularis internal rotation moment arms. The conjoint tendon may scar to the lower portion of the subscapularis, and further research is also required to investigate the biomechanical effect this has on subscapularis function.

Several limitations ought to be considered when interpreting the results of this study. While the glenohumeral joint was centered, held congruent, and its position radiographically verified during testing, some joint translations may have influenced calculations of muscle lines of action and moment arms, particularly for ABER and apprehension where the glenohumeral joint had a greater tendency to translate anteriorly and inferiorly. The glenohumeral joint center of rotation was also modeled using the center of a circle fitted to the humeral head in each plane, and in practice during dynamic movements, the axis of rotation may change, which would influence muscle moment arms. In addition, this study did not explore denervation risk, and this ought to be considered in the chosen split level. Because the upper subscapularis nerve supplies the superior half of the muscle, and the lower subscapularis nerve the inferior half, upper- and lower-third splits may present a risk of muscle denervation. Finally, the actual force generated by the conjoint tendon remains unknown, and in the present study, a variety of physiologically relevant loading scenarios were subsequently used to illustrate the relative changes that occur from no loading to maximum tendon loading.

## Conclusion

Latarjet surgery deforms subscapularis muscle fibers below the level of the split, changing muscle leverage and line of force. The midlevel subscapularis split in the Latarjet procedure confers greater mechanical advantage in terms of shoulder depressor function and stabilizing potential than that associated with an upper-third or lower-third split, particularly in positions of shoulder instability.

## Supplemental Material

sj-pdf-1-ojs-10.1177_23259671251329516 – Supplemental material for The Influence of Subscapularis Muscle Split Location on Subscapularis Function After the Latarjet Procedure Supplemental material, sj-pdf-1-ojs-10.1177_23259671251329516 for The Influence of Subscapularis Muscle Split Location on Subscapularis Function After the Latarjet Procedure by Natalia Belotti, Aaron S. Fox, Janina Henze, Richard S. Page, Lukas Ernstbrunner and David C. Ackland in Orthopaedic Journal of Sports Medicine
