# Supplementary material for: The Influence of Subscapularis Muscle Split Location on Subscapularis Function After the Latarjet Procedure
Source: Orthop J Sports Med. 2025 Apr 14;13(4):23259671251329516. doi: 10.1177/23259671251329516 (PMC12033652; doi:10.1177/23259671251329516)
Supplement: sj-pdf-1-ojs-10.1177_23259671251329516 – Supplemental material for The Influence of Subscapularis Muscle Split Location on Subscapularis Function After the Latarjet Procedure [file sj-pdf-1-ojs-10.1177_23259671251329516.pdf]

APPENDIX

Table A1: Scapular plane lines of action (degrees) for the subscapularis, including its superior, mid-superior, mid-inferior and inferior sub-regions in the case of an upper-third, mid-level and lower-third split. Data are provided for 0º abduction, 90º abduction, abduction and external rotation (ABER) and apprehension positions for the shoulder after Latarjet surgery when 0N, 20N and 40N of conjoint tendon loading is applied. Mean and standard deviation (parentheses) are provided, and significant differences in lines of action as a result of joint position, conjoint tendon load, and split level are also indicated

Muscle sub-region	Split level	0° Abduction			90° Abduction			ABER			Apprehension			Split	Load	Joint Position
		0	20	40	0	20	40	0	20	40	0	20	40			
Superior	Upper-third	178.3 (16.1)	180.3 (14.2)	177.4 (13.4)	182.6 (10.7)	182.5 (13.6)	182.9 (11.8)	169.4 (9.8)	166.6 (13.2)	170.0 (12.4)	171.2 (11.6)	169.5 (14.4)	170.7 (12.1)	0.128	0.991	< 0.001
	Mid-level	183.5 (12.1)	184.3 (11.7)	184.8 (11.7)	177.8 (22.9)	177.9 (22.2)	178.0 (21.9)	164.2 (14.1)	165.6 (14.1)	163.6 (15.1)	167.9 (12.0)	171.8 (10.4)	168.4 (12.5)			
	Lower-third	168.3 (22.5)	166.5 (21.4)	167.9 (21.8)	179.3 (14.6)	179.9 (12.9)	180.0 (12.4)	170.6 (7.4)	168.0 (6.5)	171.0 (9.1)	167.0 (10.5)	166.3 (13.1)	167.4 (12.9)			
Mid-superior	Upper-third	171.6 (13.4)	166.5 (21.8)	168.8 (24.4)	173.6 (13.5)	173.0 (15.3)	164.7 (26.9)	152.5 (16.0)	151.4 (21.5)	146.9 (24.5)	158.8 (15.3)	159.4 (18.5)	159.3 (19.8)	0.224	0.773	< 0.001
	Mid-level	170.9 (13.4)	173.5 (11.8)	173.1 (13.5)	169.6 (17.7)	169.9 (17.3)	169.8 (17.1)	159.0 (12.1)	157.8 (13.7)	158.7 (12.7)	163.5 (10.5)	165.5 (10.1)	163.0 (11.9)			
	Lower-third	165.9 (14.3)	166.7 (11.5)	166.9 (11.7)	168.8 (11.0)	167.9 (12.9)	168.2 (10.6)	158.4 (8.1)	156.5 (6.7)	160.7 (7.3)	155.1 (11.1)	169.1 (39.3)	157.0 (9.9)			
Mid-inferior	Upper-third	158.9 (14.3)	154.6 (13.1)	154.3 (17.2)	161.6 (9.5)	155.9 (13.7)	151.6 (20.1)	138.9 (14.9)	134.0 (18.5)	134.2 (17.4)	137.7 (19.5)	136.0 (19.9)	136.2 (19.1)	0.016	0.172	< 0.001
	Mid-level	160.9 (7.6)	159.7 (16.2)	158.5 (19.8)	152.7 (22.4)	151.2 (24.6)	150.3 (24.6)	139.2 (16.0)	132.5 (13.9)	129.2 (14.6)	138.8 (14.8)	136.8 (16.4)	133.0 (12.9)			
	Lower-third	157.5 (12.4)	157.8 (9.2)	157.6 (11.3)	166.8 (34.5)	156.8 (12.6)	154.8 (12.4)	144.8 (7.1)	142.4 (6.6)	145.4 (8.1)	143.7 (12.0)	145.9 (11.8)	145.2 (11.8)			
Inferior	Upper-third	151.0 (9.5)	147.0 (9.6)	146.5 (15.0)	159.9 (21.4)	146.8 (13.0)	144.7 (13.8)	136.7 (12.3)	133.4 (14.4)	133.8 (17.1)	140.4 (13.7)	140.3 (14.7)	141.7 (16.0)	0.001	0.014	< 0.001
	Mid-level	154.8 (7.2)	152.8 (6.2)	153.2 (8.1)	150.8 (12.7)	146.9 (16.3)	145.9 (17.7)	141.5 (12.3)	133.8 (16.2)	130.3 (14.9)	143.7 (13.4)	142.2 (17.5)	140.0 (15.0)			
	Lower-third	146.1 (16.7)	145.6 (14.3)	144.6 (15.1)	148.1 (11.3)	144.3 (13.6)	140.1 (17.2)	134.0 (15.0)	128.1 (15.0)	124.5 (16.4)	136.4 (16.5)	129.3 (16.9)	126.5 (17.4)			

Table A2: Adduction moment arms (mm) for the subscapularis, including its superior, mid-superior, mid-inferior and inferior sub-regions in the case of an upper-third, mid-level and lower-third split. Data are provided for 0º abduction, 90º abduction, abduction and external rotation (ABER) and apprehension positions for the shoulder after Latarjet surgery when 0N, 20N and 40N of conjoint tendon loading is applied. Mean and standard deviation (parentheses) are provided, and significant differences in lines of action as a result of joint position, conjoint tendon load, and split level are also indicated

Muscle sub-region	Split level	0° Abduction			90° Abduction			ABER			Apprehension			Split	Load	Joint Position
		0	20	40	0	20	40	0	20	40	0	20	40			
Superior	Lower-third	14.1 (11.3)	14.1 (12.5)	12.1 (11.8)	8.4 (13.6)	8.4 (13.5)	7.2 (13.2)	16.7 (9.4)	17.4 (8.8)	15.9 (10.5)	18.8 (8.8)	18.6 (10.6)	18.2 (9.2)	0.156	0.972	< 0.001
	Mid-level	8.0 (13.3)	8.3 (13.7)	7.9 (13.4)	10.5 (17.2)	9.9 (17.7)	10.3 (16.9)	21.9 (7.3)	21.6 (7.4)	22.4 (7.1)	21.4 (5.8)	20.0 (4.8)	22.0 (5.4)			
	Upper-third	8.6 (18.6)	7.0 (18.1)	11.7 (13.5)	6.6 (10.1)	3.8 (11.4)	3.8 (11.3)	17.8 (8.1)	19.0 (10.9)	17.1 (10.0)	16.2 (9.7)	16.4 (9.2)	17.0 (9.6)			
Mid-superior	Lower-third	19.0 (6.9)	9.5 (9.2)	8.9 (9.7)	7.9 (11.0)	8.2 (11.1)	7.3 (10.7)	18.3 (5.9)	18.4 (6.7)	17.7 (6.0)	20.1 (8.4)	15.4 (13.6)	19.0 (6.9)	< 0.001	0.662	< 0.001
	Mid-level	5.4 (10.9)	4.4 (11.4)	3.9 (11.7)	8.4 (15.5)	7.6 (15.4)	8.0 (14.8)	20.1 (6.6)	20.6 (7.7)	20.2 (6.8)	18.5 (5.5)	17.9 (4.9)	19.4 (5.5)			
	Upper-third	2.0 (14.7)	0.4 (15.8)	-1.1 (15.8)	2.5 (12.2)	-2.3 (16.9)	0.1 (20.0)	19.2 (7.5)	18.1 (10.1)	14.0 (14.9)	14.1 (9.1)	12.7 (8.8)	12.4 (10.7)			
Mid-inferior	Lower-third	1.4 (9.8)	-0.6 (10.4)	-1.3 (11.2)	-0.9 15.8/7	2.0 (15.1)	2.3 (14.5)	17.6 (5.6)	17.0 (7.2)	15.8 (8.7)	17.0 (8.4)	16.2 (7.8)	15.7 (7.9)	0.148	0.719	< 0.001
	Mid-level	0.2 (17.1)	-1.3 (18.3)	-1.4 (18.4)	-2.9 (10.6)	-6.8 (14.7)	-7.1 (15.5)	16.8 (3.5)	17.4 (3.3)	15.9 (3.5)	12.6 (5.9)	10.7 (6.0)	10.6 (6.4)			
	Upper-third	-0.3 (12.0)	0.0 (11.4)	-0.7 (11.7)	0.5 (13.5)	-0.2 (14.6)	0.1 (15.9)	17.5 (5.3)	17.0 (5.7)	15.0 (6.8)	12.1 (7.4)	11.7 (6.9)	11.0 (7.4)			
Inferior	Lower-third	-7.1 (4.5)	-8.0 (2.8)	-8.3 (3.0)	-4.6 (11.6)	-5.2 (11.5)	-5.3 (11.6)	7.8 (10.5)	3.6 (12.9)	4.2 (13.2)	3.4 (10.2)	2.3 (10.0)	2.5 (10.5)	0.787	0.268	< 0.001
	Mid-level	-10.1 (6.6)	-11.1 (4.5)	-12.3 (5.1)	-5.3 (10.3)	-5.9 (11.1)	-6.2 (11.8)	9.5 (5.9)	10.0 (4.9)	8.6 (4.9)	3.8 (7.4)	3.4 (7.8)	1.4 (6.8)			
	Upper-third	-5.5 (7.4)	-6.1 (6.1)	-9.5 (3.3)	-7.4 (11.2)	-7.4 (9.7)	-7.9 (9.7)	8.4 (5.0)	7.2 (5.8)	4.6 (6.9)	1.1 (5.2)	0.3 (5.3)	-1.1 (5.9)			
